# L-AVATeD: The lidar and visual walking terrain dataset

**DOI:** 10.3389/frobt.2024.1384575

**Published:** 2024-12-04

**Authors:** David Whipps, Patrick Ippersiel, Philippe C. Dixon

**Affiliations:** ^1^ Département d’informatique et de recherche opérationnelle, Université de Montréal, Montréal, QC, Canada; ^2^ Mila, the Quebec Artificial Intelligence Institute, Montréal, QC, Canada; ^3^ School of Kinesiology and Physical Activity Sciences, Université de Montréal, Montréal, QC, Canada; ^4^ Department of Kinesiology and Physical Education, McGill University, Montréal, QC, Canada

**Keywords:** artificial intelligence, environment recognition, computer vision, wearable technology, assistive robotics, gait, 3D, lidar

## 1 Introduction

Worldwide, a significant number of individuals depend on assistive robotic technologies, such as sophisticated lower-limb prostheses and comprehensive exoskeletons to address challenges related to gait and mobility (Laschowski et al., 2020; Hu et al., 2018). These innovative devices significantly improve the quality of life for people with various disabilities, enabling them to navigate real-world environments more effectively and with greater independence. Despite significant advancements in recent decades, these systems often struggle with prompt and accurate responses to changes in the local environment Hu et al. (2018). Typically, users either control these systems directly or use semi-autonomous modes, frequently needing to manually switch locomotion modes to adapt to different terrains (Laschowski et al., 2022). This requirement imposes an additional cognitive burden, potentially leading to distraction or injury. Recent research highlights the potential for substantial enhancements in exoskeleton control systems by transitioning away from user-initiated to automated locomotion mode changes (such as shifting from walking on flat surfaces to climbing stairs) to more autonomous control systems. A critical aspect of such systems is their ability to precisely classify the local environment Laschowski et al. (2020)).

Conventionally, the study and diagnosis of gait conditions is conducted in laboratory settings, where clinicians can control numerous variables and observe patients on a known walking surface. While this ensures accuracy, it limits the evaluation of gait patterns in varied natural environments and walking terrains Adamczyk et al. (2023).

Emerging studies, (e.g., Dixon et al., 2018) have demonstrated that gait is materially affected by the walking surface. Specifically, walking surfaces have been shown to impact lower-extremity muscle activity (Voloshina and Ferris, 2015), joint kinematics (Dixon et al., 2021), inter-joint coordination and variability (Ippersiel et al., 2021; Ippersiel et al., 2022), and joint kinetics (Lay et al., 2006). Freeing researchers from the constraints imposed by the laboratory requires moving away from measurement systems designed to operate in a fixed environment, towards more portable hardware solutions that can function in ecological contexts. One issue that remains, however, is the ability of portable systems to accurately classify walking surfaces.

Portable gait analysis systems with multiple participant-mounted inertial measurement units (IMUs) can themselves be used to classify walking terrain. Shah et al. (2022) used six sensors to distinguish nine surface types in a group of 30 young healthy adults using a machine learning algorithm. It remains unclear however if terrain classification accuracy from IMU data would be affected by patient pathology. That is, algorithms based on IMU data alone may incorrectly assess patient pathology or severity of impairment due to particularities of terrain on which walking is performed. The data IMUs produce is valuable, but data collection outside of the lab could cause them to suffer from exactly the problem they are trying to solve; namely, the lack of a known, controlled walking surface. While these mobile systems capture patient gait characteristics, they must also leverage additional sensor capabilities to simultaneously deliver an accurate, ground-truth classification of the local walking terrain.

Advancements in Deep Learning, particularly in Convolutional Neural Network (CNN) architectures, have shown remarkable success in image classification Krizhevsky et al. (2012). Various studies (Laschowski et al., 2022; Diaz et al., 2018; Diaz et al., 2018) have successfully used deep learning and visual data for accurate terrain identification, but these techniques require expansive data sets for their training. Combining multiple data modalities, such as depth sensors (Zhang et al., 2011; Zhang et al., 2019) and IMUs (Shah et al., 2022) show promise, though more comprehensive studies (e.g., Zhang et al., 2011), and publicly available datasets remain scarce. While databases hosting visual images or depth data are available, few if any exist in this domain that provide both modalities of data captured of a single scene (Laschowski et al., 2020). Further, none could be found that combine these modalities of data with simultaneous measurement of the device orientation sensors.

The proliferation of interpreted (stereo camera) or directly measured (LiDAR) depth data into a modern smartphone’s sensor capabilities presents a new opportunity for terrain classification. Gyroscopes, magnetometer (compass), and accelerometer sensors are now included in even modest smartphones and allow capture of device orientation and inertia at the same instant as image and depth data. This synergy of visual, depth, and orientation data can potentially enhance environmental recognition accuracy.

This paper presents a method to capture a high-resolution, multi-modal dataset in real-time without expensive, professional grade equipment, and an novel dataset that can be used in the aforementioned domains. We expect that providing access to a dataset that hosts multiple modalities of simultaneously captured data will prove invaluable to many groups, from engineers developing high level control systems for exoskeletons, to researchers studying gait. Whether using visual, depth, or other modalities of data, terrain classification systems based on deep learning techniques require voluminous training data to produce models that are accurate and generalize well.

We therefore present *L-AVATeD: the Lidar And Visual wAlking Terrain Dataset*, a novel, open-source database of visual and LiDAR image pairs of human walking terrain with simultaneously captured device orientations.

In this data report, we provide a detailed description of the dataset, our hardware and methods for collecting the data, and post-processing steps taken to improve the utility and accessibility of the data. We conclude with suggestions for how other researchers may use this dataset. Analyses of the database for walking terrain classification will be presented in future work.

## 2 Methods

### 2.1 Selection and definition of terrain types

To accurately reflect surfaces common in the built environment inside and surrounding typical North-American academic and healthcare institutions, nine terrain classes were chosen for this investigation: banked-left, banked-right, irregular, flat-even, grass, sloped-up, sloped-down, stairs-up and stairs-down.

While the class names were designed to be as descriptive as possible, some explanation is warranted.

The banked-[left, right] labels were applied in cases where the terrain declined significantly, perpendicular to the direction of motion. In cases where another class might also apply (e.g., grass, irregular), these labels were given precedence.

The flat-even class was a base-case; indicating any terrain that was generally smooth and solid, and neither sloped, banked nor grassy. Samples may include any material (e.g., concrete, tile) or color (there are many examples exhibiting bright colours and patterns).

Irregular surfaces were defined as those that had no slope up/down or left/right and had enough irregularity that they might be expected to materially affect gait. Examples include cobblestone, gravel, and rough mud.

Surfaces were labelled as *grass* if they were generally flat, similar to irregular in that they should be neither sloped nor banked, and consisted primarily of short grasses found on typical found in North American lawns.

sloped-[up,down] were defined as any surface (including grassy ones) which had a significant incline or decline in the immediate direction of motion.

stairs-[up,down] were the easiest to label, and consisted of stairs of any material, indoors or out.

**FIGURE 1:**
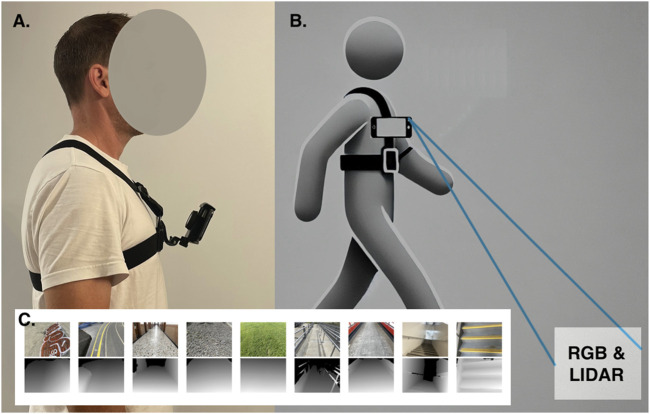
**(A)** Data collection harness with iPhone sensor rig. **(B)** Approximate data capture field. **(C)** Example dataset image pairs by class. RGB (top), LiDAR (bottom) from left: banked-left, banked-right, flat-even, irregular, grass, sloped-down, sloped-up, stairs-down, stairs-up.

Examples of visual and LiDAR image from each class can be seen in Figure 1.

### 2.2 Data-collection hardware

Apple’s (Apple Inc., Cupertino, USA) iPhone (iOS) was chosen as the platform for collecting our walking terrain dataset due to their built-in LiDAR sensor as well as extensive, well-documented APIs for capturing and manipulating depth data. Data were captured on three physical devices (1 iPhone 12 Pro and two iPhone 14 Pros). While it is possible to extract depth data from mobile devices which interpret depth data using stereoscopic methods Wang et al. (2019), we chose devices which contain a built-in LiDAR scanner, for accuracy and consistency.

Visual (RGB) images were captured in landscape orientation using the front-facing cameras of each of the iPhone 12 and 14. Data were captured at the native resolution of the sensor: 4,032 × 3,024 pixels, RGB, 8-bit per channel and were stored using the Apple High Efficiency Image File Format (HEIF).

The specifications for the LiDAR scanner are not available directly from the manufacturer, but Luetzenburg et al. (2021) performed and in-depth analysis of the iPhone 12/14 LiDAR hardware (the devices share the same sensor) for applications in geosciences. Their conclusions coincide with the output LiDAR depth map dimensions we recorded, at 768 × 576 pixels. In this dataset, each LiDAR “pixel” uses a full 32-bits to store depth data.

It should be noted that while differing device models were used to collect samples, both the visual and LiDAR data were identical in spatial and depth resolution.

### 2.3 Data collection

Data were collected over 6 months during the spring and summer of 2023, by three young healthy adult members of our research lab. Participants were fitted with a chest-mounted mobile-phone harness. The harness allowed for hands free data capture and provided some consistency in data capture across participants. iPhones were clipped to the harness horizontally (i.e., in “landscape orientation”) using the built-in mount, with an initial angle between 30–50° downward from the horizon. This orientation provided a wide view of the local walking surface within about a meter in front of the participant and up to roughly 5 m away, depending on the local terrain. The variation in mounting angle was similar in magnitude to the small up and down variation of camera field of view introduced by simple act of walking. This small amount of noise acts, in effect, as a natural regularizer for the dataset, and will help, e.g., a Convolutional Neural Network trained on it to better generalize on unseen data.

A custom iOS application was written to simplify simultaneous capture and labelling of visual, LiDAR, and device orientation data at 1 Hz. This capture frequency provided a balance between volume of data recorded while preventing too many captures of the same visual scene (i.e., walking terrain). In an individual capture session, participants would select the terrain type (based on their visual interpretation upcoming terrain and the definitions above) in the capture application, tap begin data capture, and terminate capture before the terrain class changed. The data would then be automatically labelled and stored on the device. Data were imported from each device into a central repository and individually reviewed (by D.W.) for labelling errors. All members who participated in data collection were briefed on use of the system prior to data collection.

Data were labelled in sequence, with a numeric prefix indicating the order of capture [000-999], and a unique suffix in the form of a universally unique identifier (UUID). Every image pair has the same file name, apart from an additional suffix “_depth” on the LiDAR disparity map and differing file extension. The gravity vector data was captured into individual comma-separated-values files, each with the matching UUID suffix.

7,968 RGB/LiDAR image pairs were captured along with the device orientation gravity vector, but due to availability of suitable terrain, class data is imbalanced (Table 1). Class imbalance, while not ideal, can be easily handled using any number of techniques when actually making use of the data. While training a Convolutional Neural Network, for example, oversampling of the minority class, adding class weights to the loss function, or using ensemble methods such as bagging and boosting are some common solutions.

**TABLE 1:** Per-class characteristics of the Lidar And Visual wAlking Terrain Dataset.

Class	Images	Proportion	Min. Pitch	Max. Pitch	Min. Roll	Max. Roll
Banked-left	466	5.8%	43.2°	70.2°	−11.4°	26.6°
Banked-right	459	5.8%	43.1°	70.7°	−23.3°	11.5°
Flat-level	2,339	29.4%	16.4°	78.9°	−18.9°	18.2°
Irregular	920	11.5%	20.5°	73.1°	−15.2°	16.1°
Grass	1,217	15.3%	17.6°	72.0°	−15.6°	17.0°
Sloped-down	510	6.4%	25.6°	73.0°	−19.4°	19.7°
Sloped-up	428	5.4%	27.7°	68.4°	−14.8°	12.4°
Stairs-down	792	9.9%	22.3°	74.9°	−28.8°	21.6°
Stairs-up	837	10.5%	17.6°	86.7°	−34.3°	19.6°

### 2.4 Post-processing

With the goal of making the dataset more manageable (the raw dataset is almost 45GBs in size), RGB images were pre-processed by reducing their size from 4032 × 3024 pixels to 512 × 384 pixels. Resizing used Apple’s Scriptable Image Processing System (SIPS) and simultaneously converted images to a more standard JPEG format from their native HEIF format.

Processing the LiDAR data required special care, as it is captured in a format not readily consumed by typical image libraries. LiDAR data were captured and stored natively as 32-bit disparity maps (disparity = 1/distance) and saved to TIFF files. These files were down sampled and normalized using OpenCV Itseez (2015), converted to 8-bit grayscale, and exported to JPEG. At 768 × 576 pixels, the spatial resolution of the built-in LiDAR scanner is much lower than the visual camera, and so these images were not pre-scaled.

A device orientation vector was also recorded at the instant both the visual and LiDAR data were captured. This orientation vector is recorded relative to gravity and the iPhone device axes. It is normalized in each direction in units of the accepted acceleration due to gravity at Earth’s surface, i.e., $9.8\;m/s^{2}$ . These vectors are difficult to use in their raw (x,y,z) vector format, and were translated to more understandable *pitch* and *roll* (Equations 1, 2 respectively) values using the *two-element arc-tangent* function:

$$pitch=atan2\left(z,x\right)$$ (1)

and,

$$roll=atan2\left(z,y\right)$$ (2)

where pitch is defined as the angle down from the horizon, and roll as deviation either clockwise or anti-clockwise from the horizon line itself.

## 3 Discussion

A comprehensive understanding of the local walking context is crucial both for human gait analysis and the advanced control systems of robotic prostheses. Relying solely on laboratory analysis limits researchers’ capacity to accurately evaluate clinical gait in patients and hinders the optimal functioning of robotic prostheses in varied ecological contexts. Consequently, conducting studies across a spectrum of walking terrains is essential to address these limitations. A ground-truth understanding of walking terrain is traditionally identified using simple visual data and Deep Neural Networks, in particular Convolutional Neural Networks (CNNs). While CNNs are ideal for this classification task, they require extensive training data which may not be readily available.

Multiple studies ((Ophoff et al., 2019; Melotti et al., 2018; AlDahoul et al., 2021)) have revealed that classifiers trained using multi-modal data (and in particular a combination of depth and visual data) can outperform simple visual classification in object-detection and image classification (Schwarz et al., 2015). Previous datasets in the context of walking terrain have provided only a single modality of data (visual), or have been limited in their terrain classes. A novel dataset providing walking terrain data, simultaneously captured in both visual and depth modalities could therefore provide huge advantages to researchers training visual classifiers (in particular CNNs) for use in these domains. The L-AVATeD dataset fills this gap, improving on existing datasets by providing researchers and engineers a baseline set of multi-class, multi-sensor, walking terrain data in multiple modalities.

L-AVATeD is notable for being the only open-source database of its kind to provide not just two, but three modalities of data captured simultaneously for a given scene of walking terrain. While not as large as the largest available walking terrain datasets reviewed by Laschowski et al. (2020), at almost 8,000 samples L-AVATeD matches the median dataset size. Further, the spatial resolution of the published RGB images in L-AVATeD (at 4032 × 3024 pixels) is more than 9 times higher than the highest resolution presented. Images at resolutions of this magnitude may prove unwieldy for most neural networks, especially the low-resource-optimized architectures available for use in mobile and edge-computation hardware. It remains important however to preserve as much signal as possible to not limit future research, and so full-resolution images are provided.

Depth data, captured via LiDAR were in fact saved as *disparity* maps (i.e., 1/distance ). Notably, signal decreases as distance increases. At 768 × 576 “pixels” of native spatial resolution, these too are the most information-dense depth measurements in any of the environment recognition systems reviewed in Laschowski et al. (2020). To ensure we did not introduce any algorithmic bias to the depth data, the capture program was instructed to replace missing signal (depth “pixels”) with a placeholder NaN value rather than automatically interpolating such points. This allows researchers to handle the missing data however they see fit, for example,: replacing with zeros, average depth, or an interpolation scheme. The raw depth maps are available as TIFF files. To make the depth data easily accessible, a copy is provided created using a very simple algorithm of replacing missing data with zeros, normalizing, and then compressing the depth dimension into 8 bits before saving the file as a JPEG. This format provides a representation suitable for use in most deep neural network software packages. It should be noted however that this simple algorithm is effectively a lossy compression of the data, and while useful, should not be considered as the most information-rich representation. Examples of these lossy depth-data representations can be seen in Figure 1 (bottom row).

Device orientation data were captured as a gravity vector. The raw data are available as comma-separated values, indexed with the sample UUID. This raw format is not easily interpreted by humans, and so each was converted to a more easily understood *pitch* (camera angle up and down relative to the horizon) and *roll* (camera rotation clockwise and anti-clockwise relative to the horizon). Per-class pitch and roll statistics are provided in Table 1. Preliminary *post hoc* analyses of these statistics do not immediately reveal significant signal, but used in combination with the RGB and LiDAR data counterparts, in a deep learning context in particular could prove fruitful. For example, researchers might use this data to “de-rotate” an image using the inverse device rotation angle before passing it into the classifier, potentially improving classifications where horizon angle of the scene may be important.

The number of samples in this dataset count almost two orders of magnitude smaller than the largest datasets available Laschowski et al. (2020). Its usefulness however lies not in its number of samples, but in the diversity of information in those samples. The fusion of multi-modal sensor data has been shown to enhance task accuracy in many domains El Madawi et al. (2019), AlDahoul et al. (2021), Gao et al. (2018). This dataset in particular will be useful in training accurate control systems for robotic prostheses Diaz et al. (2018), locomotion modes for wearable robotics Li et al. (2022), and mobile gait analysis systems. More specifically, a deep neural network combining multiple CNNs (for visual and depth data) modulated by device pitch and roll values could be trained to accurately classify terrain in real time Laschowski et al. (2022).

These data have been made available through IEEE DataPort, and users of the L-AVATeD are requested to reference this report.

## Data Availability

The datasets presented in this study can be found in online repositories. The names of the repository/repositories and accession number(s) can be found below: https://ieee-dataport.org/documents/l-avated-lidar-and-visual-walking-terrain-dataset.
